# A theoretical model for roller-shape design of three-roller continuous and synchronous calibration process of ovality and straightness for large thin-walled pipes

**DOI:** 10.1038/s41598-024-57753-0

**Published:** 2024-03-26

**Authors:** Xueying Huang, JinPing Gu

**Affiliations:** 1 School of Digital Technology and Engineering, Ningbo University of Finance and Economics, Ningbo City, 315175 People’s Republic of China; 2 Ministry Education, Key Lab Mech Theory & Equipment Design, Tianjin University, Tianjin, 300350 People’s Republic of China

**Keywords:** Large thin-walled pipes, Calibration process of ovality and straightness, Theoretical model, Roller-shape design, Ovality, Straightness, Ocean sciences, Engineering

## Abstract

Large thin-walled pipes are particularly suitable for oil and gas transport and auxiliary pipes in cold areas or deep sea-beds. At present, the rounding and straightening processes are completed independently, and the theoretical model for roller-shape design is analyzed only in a single direction. To solve this problem, a theoretical model for roller-shape design of three-roller continuous and synchronous adjusting straightness and ovality process for large thin-walled pipes is established. The established model is verified by numerical simulation and experimental research using 304 stainless steel pipes. The results show that the three roller-shape design schemes, including three-section, four-section and five-section, proposed based on the theoretical model, can obtain qualified formed pipes. Based on the model, the residual ovality, residual straightness and maximum residual stress of the three roller-shape schemes are discussed. The residual straightness can reach within 0.2%, and the residual ovality can reach within 1%. It verifies the applicability of the model and the feasibility of the process. The model can provide a theoretical basis for presetting the process parameters and optimizing the roller-shape.

## Introduction

Large thin-walled pipes are widely used in oil, natural gas, aerospace and power industries. The most prominent of which is the construction of oil and gas pipelines. Natural gas has become the main energy in the world. Large thin-walled pipes are widely used in oil and gas transportation in cold zones or deep water. API Spec 5L (Line pipe Specification developed by the American Petroleum Institute’s Fifth Committee) has strict requirements for the straightness and ovality of finished welded pipes^1^. Affected by factors such as welding thermal stress, material properties and forming equipment, the straightness and ovality of the formed welded pipe cannot meet the industry standard. It needs to be straightened and rounded again. Expanding the pipe diameter, increasing the pressure and improving the steel grade are the development trends to further improve the transportation capacity of pipelines. The accuracy of the dimension of large thin-walled pipes is required to be higher and higher. Currently, the existing straightening and rounding processes are done independently. As a result, it is not easy to realize automated and intelligent production. On the other hand, a single roller-shape cannot solve the flattening problem of large thin-walled pipes. It is difficult to adjust the straightness and ovality to the optimal level at the same time. It seriously affects the on-site welding and pipeline safety. Therefore, the study of the roller-shape theoretical model to realize the cooperative control of straightness and ovality has become a key technology for the production of large thin-walled pipes. It is also a bottleneck problem restricting the construction of oil and gas pipelines.

The existing roller-shape design studies are only applicable to the straightening process. Roller-shape designs that combine both straightening and rounding have not yet appeared. Most of the research on roller-shape designs by researchers focuses on the engineering application. Its application scope is limited. Moreover, there is a lack of theoretical guidance on roller-shape design. It is difficult to carry out systematic research.

Equal curvature split die or equal curvature roller are adopted to realize the rounding process during the rounding process. The rounding process mainly includes: whole-diameter rounding, over-bending rounding and roller rounding. The production of large thin-walled pipes is usually rounded by adjusting a whole-diameter^2,3^. However, the method will change the circumference of pipes. When the size of circumference is qualified, the method is not applicable. Based on the above situation, Zhao et al., proposed the over-bending rounding process. A pair of equal curvature upper and lower flap dies with small curvature were used to apply pressure to pipes along the long axis. It made the long axis shorter and the short axis longer, and produced elastoplastic deformation to achieve the rounding^4^. This process is not suitable for the overall rounding of pipes. The roller rounding is a process in which the position of the pipe changes by the rotational motion of a roller of equal curvature^5^. As for the roller rounding, Yu et al.^6–8^ proposed a three-roller rounding process. The upper roller is placed inside the pipe, which requires a cylinder turning device during the feeding process. However, when the length of the pipe is relatively long, the stiffness of the upper roller is not enough. In view of this, Huang et al.^9,10^ proposed a rounding process in which three rollers are placed on the outside of pipes. This process can realize the overall and continuous rounding of pipes. It effectively reduces the residual stress and improve the quality of pipes.

To obtain high-quality, high-precision pipes, researchers have carried out a lot of research in the design of straightening rollers. The straightening process mainly includes pressure straightening and oblique roller straightening. Song et al.^11,12^ proposed two straightening control strategies for large longitudinal welded pipes based on the three-point bending principle. The spacing between the fulcrums is a little big. So, there is a big deflection after deformation. Oblique roller straightening is used for pipes straightening. That is, based on three-step bending, the single-point bending straightening is converted into uninterrupted compression bending straightening. For the oblique roller straightening, Ma et al.^13^ proposed a variable curvature roller-shape design, which was verified by theoretical analysis methods. Wang et al.^14^ established a model of a ten-roller straightener by using hyperbolic roller shape. Yi et al.^15,16^ set up a multi-roller wave straightening model and analyzed the influence of roller parameters on the straightening process. Ma et al.^17^ built a segmented straightening roller and proved the effectiveness of the method. In summary, it can be seen that the design of the straightening roller plays a decisive role in the straightening process. Having a proper roller is the key to increasing straightening productivity and producing high-quality products. Based on this, Huang et al. proposed a “segmented” roller design for the first time. The proportion of the roller area, the curvature design, the elastic area ratio, and the feasibility of the process were studied^18^.

In this paper, a theoretical model of roller-shape design is established. This model is for the characteristics of large thin-walled pipes. It is suitable for continuous and synchronous calibration process of ovality and straightness with rollers. This model provides a theoretical basis for the setting of process parameters and the optimization of roller shapes. Based on the traditional assumptions, this paper establishes the curvature equations of the roller. Based on the obtained curvature equations, the springback curvature equations of the circumferential and axial directions of the pipe are established. According to the theoretical model and the design idea of “segmented”, three roller-shape schemes of “Three-section”, “Four-Section” and “Five-Section” are proposed. Numerical simulation analysis is carried out on the above schemes. The effects of the three roller-design schemes on the residual straightness, residual ovality and maximum residual stress are studied. The theoretical model is verified by numerical simulation. Additionally, through physical experiments, the feasibility of the “Five-Section” roller-shape scheme is carried out.

## Process introduction

**Figure 1:**
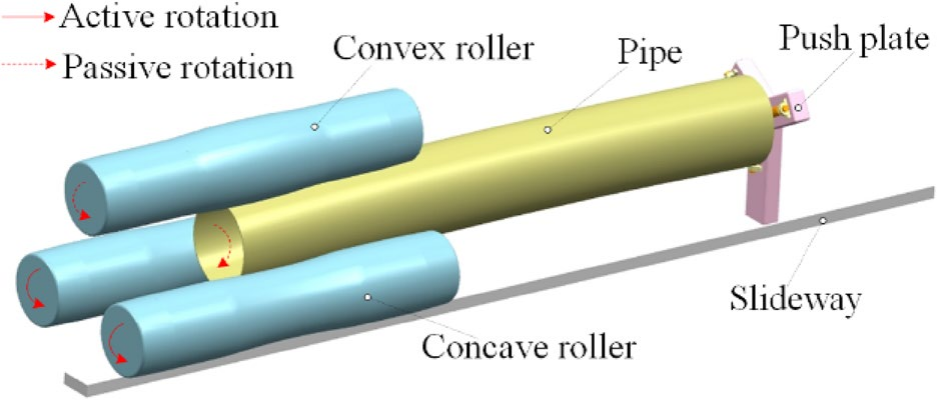
Schematic diagram of process.

As shown in Fig. 1, the main working parts of this process are three parallel rollers, including a convex roller (upper roller) and two concave rollers (lower roller). Two concave rollers are driven by servo motors to rotate simultaneously, while the pipe and convex rollers are driven to rotate under friction. At the same time, the pipe is continuously pushed along the slideway through the push plate to achieve the calibration process.

**Figure 2:**
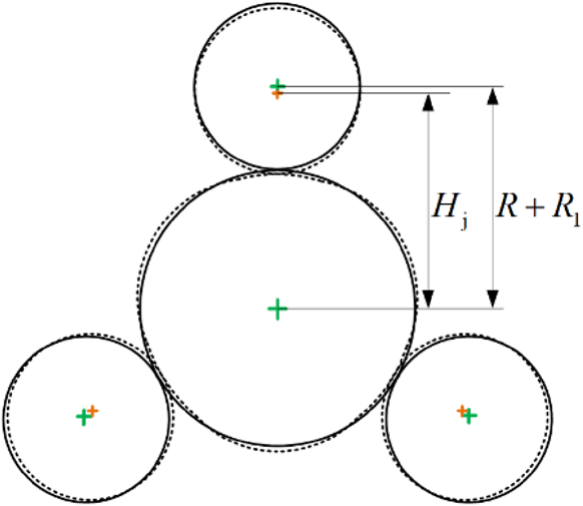
Diagram of loading parameters.

As shown in Fig. 2, the three rollers move synchronously towards the center of the pipe with the same radial reduction. The radial reduction of each roller is recorded as *H*.

1 $$H = R_{1} + R - H_{{\text{j}}}$$

where $H$ is the radial reduction; ${R}_{1}$ is the radius of roller; $R$ is the radius of pipe; ${H}_{j}$ is the distance from the center of pipe to the center of roller after loading.

## Theoretical model of roller-shape design

### Basic assumptions

1. The pipe is continuous, homogeneous, and isotropic. The linear simple kinematic hardening (LSKH) constitutive model^19^ is adopted, as shown in Fig. 3.
  2 $$\begin{aligned} \sigma & = \left\{ \begin{gathered} D\varepsilon + \sigma_{0} \begin{array}{*{20}c} {} & {\varepsilon > 0} \\ \end{array} \hfill \\ D\varepsilon - \sigma_{0} \begin{array}{*{20}c} {} & {\varepsilon < 0} \\ \end{array} \hfill \\ \end{gathered} \right. \\ \sigma_{0} & = \left( {1 - \frac{D}{E}} \right)\sigma_{{\text{s}}} \\ \end{aligned}$$
  where ${\sigma }_{s}$ is yield stress; $D$ is plastic modulus; $E$ is elastic modulus; $\sigma$ is the stress; $\varepsilon$ is the strain; $\sigma_{0}$ is the intercept stress.

**Figure 3:**
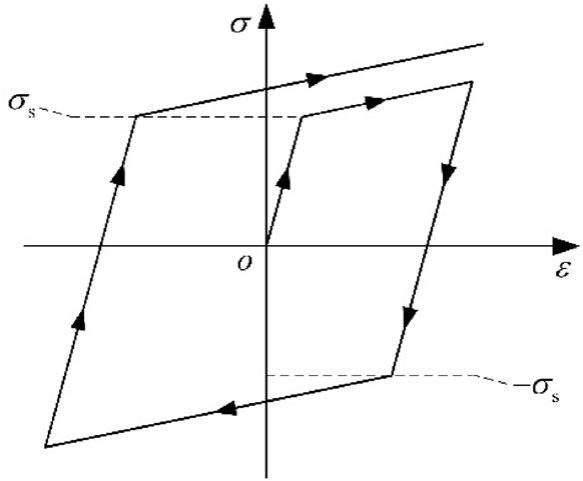
LSKH constitutive model.
2. Any cross-section of the pipe is always perpendicular to the geometric central axis, remains a plane during the deformation. There is no tilt or distortion between the two adjacent cross-sections^20^. So, the shear stress and shear strain are negligible.
3. The deformation of pipe follows the principle of volume invariance.
4. According to the theory of thin-walled shells, the change of wall thickness is ignored, namely ${\varepsilon }_{r}=0$.
5. Because the movement of neutral layer is small for thin-walled pipe in the deformation process, it can be considered that the strain neutral layer, stress neutral layer and geometric central layer of the pipe are always coincided.

### Roller meshing curve equation

**Figure 4:**
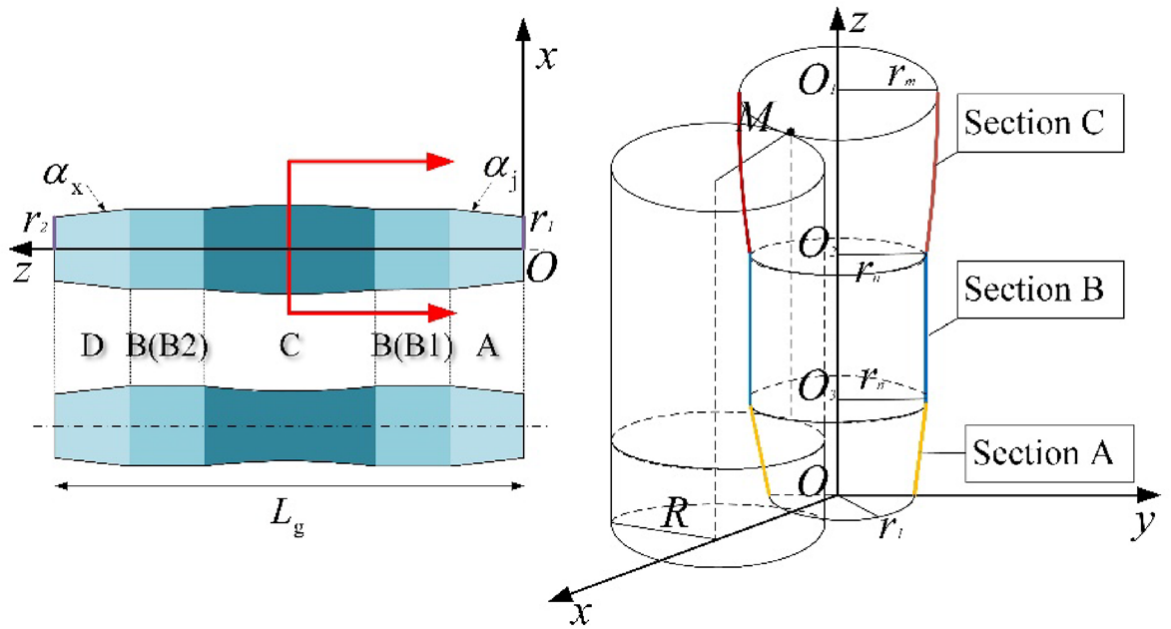
Schematic diagram of the “Five-section” roller shape (On the right side of this figure is a one-half convex roller). A, Loading section; B (B1 and B2), ovality calibration section; C, ovality and straightness calibration section; D, unloading section.

The schematic diagram of roller shape is shown in Fig. 4. A is the loading section, that is, the inlet end. To make the pipe enter the gap between three rollers and achieve radial reduction, it is designed to be truncated cone shape. D is the unloading section, that is, the outlet end. Its shape is also designed to be truncated cone to ensure that the pipe can be smoothly unloaded after calibration. B1 and B2 are the ovality calibration sections, where the shape is cylindrical. C is the synchronous calibrating straightness and ovality section.

Establish the Cartesian coordinate system *OXYZ*. Let the roller and the pipe mesh at point *M*, and point *M* is (*x, y, z*) at the *OXYZ* coordinates. Since the pipe rotates between three rollers, any point on the surface of the pipe alternately undergoes variable curvature. The point *M* is always in line with the change in curvature on the roller.

Let the total length of the roller be *L*_g_. Taking the “Five-section” roller as an example, the proportional parts of the loading section, the ovality calibration section, the ovality and straightness calibration section, the supplementary ovality calibration section and the unloading section of the roller are *n*_1_: *n*_2_: *n*_3_: *n*_4_: *n*_5_ respectively.

#### Loading section (section A)

3 $$\alpha_{\text{j}} = \frac{{3(2H_{\max } + 2a - D_{\text{P}} )}}{{L_{\text{g}} }}$$

According to the shape diagram of the roller, it can be seen that,

4 $$\alpha_{\text{j}} = \frac{{2(r_{{\text{n}}} - r_{1} )}}{{ln_{1} }}$$

where, $l = \frac{{L_{\text{g}} }}{{{\text{n}}_{1} + {\text{n}}_{2} + {\text{n}}_{3} + {\text{n}}_{4} + {\text{n}}_{5} }}$ is the length of each part; ${r}_{n}$ is the radius at the intersection of sections B and A; ${r}_{1}$ is the minimum radius of section A.

In the *OXZ* plane of the Fig. 4, set the curve equation of the section A as

5 $$x = kz + b(k \ne 0)$$

where, *b* is the coefficient of the primary equation. Based on the geometric characteristics of section A, the boundary conditions can be obtained,

6 $$\left\{ \begin{gathered} z = 0\begin{array}{*{20}c} \to & {b = r_{1} } \\ \end{array} \hfill \\ {\text{z}} = {\text{l}} {\text{n}}_{1} \begin{array}{*{20}c} \to & {x = r_{{\text{n}}} } \\ \end{array} \hfill \\ \end{gathered} \right.$$

Substituting the boundary conditions into Eq. (5),

7 $$k{ = }\frac{1}{2}\alpha_{{\text{j}}} \begin{array}{*{20}c} {;} & {b = r_{1} = r_{{\text{n}}} } \\ \end{array} - \frac{1}{2}\alpha_{{\text{j}}} \ln_{1}$$

So, the curve equation for section A is,

8 $$x = \frac{1}{2}\alpha_{{\text{j}}} z + \left( {r_{{\text{n}}} - \frac{1}{2}\alpha_{{\text{j}}} ln_{1} } \right)\begin{array}{*{20}c} {} & {\left( {0 \le z \le ln_{1} } \right)} \\ \end{array}$$

#### Ovality calibration section (section B)

In the *OXZ* plane of the Fig. 4, the section B is a straight segment with a curvature of *k* = 0. The curve equation for the section B is:

9 $$x = kz + b(k = 0)$$

Based on the geometric characteristics of section B, the boundary conditions can be obtained,

10 $$b = r_{{\text{n}}}$$

By substituting the boundary conditions into Eq. (9), the curve equation for section B can be obtained as

11 $$x = r_{{\text{n}}}$$

$$ln_{1} \le z \le l\left( {n_{1} + n_{2} } \right);l\left( {n_{1} + n_{2} + n_{3} } \right) \le z \le l\left( {n_{1} + n_{2} + n_{3} + n_{4} } \right)$$

#### Ovality and straightness calibration section

In the *OXZ* plane of the Fig. 4, the curve equation of the section C is hyperbolic^18^.

12 $$\frac{{x^{2} }}{{a^{2} }} - \frac{{z^{2} }}{{b^{2} }} = 1$$

where, *a* and *b* are the coefficients of the hyperbolic equation. Based on the geometric characteristics of section A, the boundary conditions can be obtained,

13 $$\left\{ \begin{gathered} z = \frac{1}{2}\ln_{3} \begin{array}{*{20}c} \to & {x = r_{{\text{n}}} } \\ \end{array} \hfill \\ {\text{z}} = 0\begin{array}{*{20}c} \to & {x = r_{{\text{m}}} } \\ \end{array} \hfill \\ \end{gathered} \right.$$

where, ${r}_{m}$ is the radius of the center position of the section C.

Substitute the boundary conditions into Eq. (12),

14 $$\left\{ \begin{gathered} a^{2} { = }r_{{\text{m}}}^{2} \hfill \\ b^{2} = \frac{{n_{3}^{2} l^{2} r_{{\text{m}}}^{2} }}{{4\left( {r_{{\text{n}}}^{2} - r_{{\text{m}}}^{2} } \right)}} \hfill \\ \end{gathered} \right.$$

Therefore, the curve equation for the section C is,

15 $$\frac{{x^{2} }}{{r_{{\text{m}}}^{2} }} - \frac{{4z^{2} \left( {r_{{\text{n}}}^{2} - r_{{\text{m}}}^{2} } \right)}}{{n_{3}^{2} l^{2} r_{{\text{m}}}^{2} }} = 1$$

By substituting the above curve equation with coordinates, it can be obtained that

16 $$\frac{{x^{2} }}{{r_{{\text{m}}}^{2} }} - \frac{{4\left( {z - l\left( {n_{1} + n_{2} + \frac{{n_{3} }}{2}} \right)} \right)^{2} \left( {r_{{\text{n}}}^{2} - r_{{\text{m}}}^{2} } \right)}}{{n_{3}^{2} l^{2} r_{{\text{m}}}^{2} }} = 1$$

Therefore, the curve equation shown can be expressed as,

17 $$\begin{aligned} x & = \sqrt {\frac{{4\left( {z - l\left( {n_{1} + n_{2} + \frac{{n_{3} }}{2}} \right)} \right)^{2} \left( {r_{{\text{n}}}^{2} - r_{{\text{m}}}^{2} } \right)}}{{n_{3}^{2} l^{2} }} + r_{{\text{m}}}^{2} } \\ l\left( {n_{1} + n_{2} } \right) & \le z \le l\left( {n_{1} + n_{2} + n_{3} } \right) \\ \end{aligned}$$

The curvature equation for section C can be expressed as,

18 $$k = \frac{{n_{3}^{4} l^{4} r_{{\text{m}}}^{5} }}{{\left( {\left( {\left( {z{ - }l\left( {n_{1} + n_{2} + \frac{{n_{3} }}{2}} \right)} \right)^{2} } \right)\left( {r_{{\text{m}}}^{2} + \frac{{n_{3}^{2} l^{2} r_{{\text{m}}}^{2} }}{{4\left( {r_{{\text{n}}}^{2} - r_{{\text{m}}}^{2} } \right)}}} \right) + \frac{{n_{3}^{4} l^{4} r_{{\text{m}}}^{4} }}{{16\left( {r_{{\text{n}}}^{2} - r_{{\text{m}}}^{2} } \right)}}} \right)^{{{3 \mathord{\left/ {\vphantom {3 2}} \right. \kern-0pt} 2}}} \left( {16\left( {r_{{\text{n}}}^{2} - r_{{\text{m}}}^{2} } \right)^{2} } \right)}}$$

#### Unloading section

19 $$\alpha_{{\text{x}}} = \frac{{{6}H_{{{\text{max}}}} }}{{L_{{\text{g}}} }}$$

where, $H_{{{\text{max}}}}$ is the maximum amount of radial reduction.

According to the shape of the roller,

20 $$\alpha_{{\text{x}}} = \frac{{2(r_{{\text{n}}} - r_{{2}} )}}{{ln_{{5}} }}$$

where, $r_{2}$ is the minimum radius of the section D.

In the *OXZ* plane of the Fig. 4, let the curve equation for the section D be,

21 $$x = kz + b(k \ne 0)$$

Based on the geometric characteristics of section D, the boundary conditions can be obtained,

22 $$\left\{ \begin{gathered} z = \left( {n_{1} { + }{\text{n}}_{{2}} { + }{\text{n}}_{{3}} { + }{\text{n}}_{{4}} } \right)l\begin{array}{*{20}c} \to & {x = r_{{\text{n}}} } \\ \end{array} \hfill \\ {\text{z}} = L_{{\text{g}}} \begin{array}{*{20}c} \to & {x = r_{{2}} } \\ \end{array} \hfill \\ \end{gathered} \right.$$

Substitute the boundary conditions into Eq. (21),

23 $$k{ = - }\frac{1}{2}\alpha_{{\text{x}}} \begin{array}{*{20}c} {;} & {b = r_{{\text{n}}} } \\ \end{array} { + }\frac{1}{2}\alpha_{{\text{x}}} {\text{l}} \left( {n_{1} { + }n_{2} + n_{3} + n_{4} } \right)$$

Therefore, the curve equation for the section D is,

24 $$\begin{aligned} x & = - \frac{1}{2}\alpha_{{\text{x}}} z{ + }{\text{r}}_{{\text{n}}} { + }\frac{1}{2}\alpha_{{\text{x}}} {\text{l}} \left( {n_{1} { + }n_{2} + n_{3} + n_{4} } \right) \\ l & \left( {n_{1} + n_{2} + n_{{3}} + n_{{4}} } \right) \le z \le L_{{\text{g}}} \\ \end{aligned}$$

In summary, the roller curve equation is,

25 $$x = \left\{ \begin{gathered} \frac{1}{2}\alpha_{{\text{j}}} z + \left( {r_{{\text{n}}} - \frac{1}{2}\alpha_{{\text{j}}} ln_{1} } \right)\begin{array}{*{20}c} {} & {\left( {0 \le z \le ln_{1} } \right)} \\ \end{array} \hfill \\ r_{{\text{n}}} \begin{array}{*{20}c} {} & {ln_{1} \le z \le l\left( {n_{1} + n_{2} } \right){;}l\left( {n_{1} + n_{2} + n_{3} } \right) \le z \le l\left( {n_{1} + n_{2} + n_{3} + n_{4} } \right)} \\ \end{array} \hfill \\ \sqrt {\frac{{4\left( {z - l\left( {n_{1} + n_{2} + \frac{{n_{3} }}{2}} \right)} \right)^{2} \left( {r_{{\text{n}}}^{2} - r_{{\text{m}}}^{2} } \right)}}{{n_{3}^{2} l^{2} }} + r_{{\text{m}}}^{2} } \begin{array}{*{20}c} {} & {l\left( {n_{1} + n_{2} } \right) \le z \le l\left( {n_{1} + n_{2} + n_{3} } \right)} \\ \end{array} \hfill \\ - \frac{1}{2}\alpha_{{\text{x}}} z{ + }{\text{r}}_{{\text{n}}} { + }\frac{1}{2}\alpha_{{\text{x}}} {\text{l}} \left( {n_{1} { + }n_{2} + n_{3} + n_{4} } \right)\begin{array}{*{20}c} {} & {l\left( {n_{1} + n_{2} + n_{{3}} + n_{{4}} } \right) \le z \le L_{{\text{g}}} } \\ \end{array} \hfill \\ \end{gathered} \right.$$

The equation for the curvature of the roller is,

26 $$k = \left\{ \begin{gathered} \frac{1}{2}\alpha_{{\text{j}}} \begin{array}{*{20}c} {\begin{array}{*{20}c} {} & {0 \le z \le ln_{1} } \\ \end{array} } & {} \\ \end{array} \hfill \\ 0\begin{array}{*{20}c} {\begin{array}{*{20}c} {} & {ln_{1} \le z \le l\left( {n_{1} + n_{2} } \right){;}l\left( {n_{1} + n_{2} + n_{3} } \right) \le z \le l\left( {n_{1} + n_{2} + n_{3} + n_{4} } \right)} \\ \end{array} } & {} \\ \end{array} \hfill \\ \frac{{n_{3}^{4} l^{4} r_{{\text{m}}}^{5} }}{{\left( {\left( {\left( {z - l\left( {n_{1} + n_{2} + \frac{{n_{3} }}{2}} \right)} \right)^{2} } \right)\left( {r_{{\text{m}}}^{2} + \frac{{n_{3}^{2} l^{2} r_{{\text{m}}}^{2} }}{{4\left( {r_{{\text{n}}}^{2} - r_{{\text{m}}}^{2} } \right)}}} \right) + \frac{{n_{3}^{4} l^{4} r_{{\text{m}}}^{4} }}{{16\left( {r_{{\text{n}}}^{2} - r_{{\text{m}}}^{2} } \right)}}} \right)^{{{3 \mathord{\left/ {\vphantom {3 2}} \right. \kern-0pt} 2}}} \left( {16\left( {r_{{\text{n}}}^{2} - r_{{\text{m}}}^{2} } \right)^{2} } \right)}}\begin{array}{*{20}c} {\begin{array}{*{20}c} {} & {l\left( {n_{1} + n_{2} } \right) \le z \le l\left( {n_{1} + n_{2} + n_{3} } \right)} \\ \end{array} } & {} \\ \end{array} \hfill \\ - \frac{1}{2}\alpha_{{\text{x}}} \begin{array}{*{20}c} {\begin{array}{*{20}c} {} & {l\left( {n_{1} + n_{2} + n_{{3}} + n_{{4}} } \right) \le z \le L_{{\text{g}}} } \\ \end{array} } & {} \\ \end{array} \hfill \\ \end{gathered} \right.$$

### Curvature after springback

It is assumed that the pipe fits perfectly with the three rollers during the process. Then, the curvature at any point on the surface of the roller coincides with the curvature of the pipe after loading.

Since the deformation of each section in the process is continuous, the initial curvature is the curvature after the last bending and springback. According to Ref.^21^, the recursive equation for reciprocating bending can be expressed as

27 $$K_{{{\text{p}}n}} = \left\{ \begin{gathered} \begin{array}{*{20}c} {K_{1} - \frac{{M_{1} (K_{1} ,K_{0} )}}{EI}} & {n = 1} \\ \end{array} \hfill \\ \begin{array}{*{20}c} {K_{n} - \frac{{M_{n} (K_{n} ,K_{{{\text{p}}(n - 1)}} )}}{EI}} & {n \ge 2} \\ \end{array} \hfill \\ \end{gathered} \right.$$

where, *n* is the number of reciprocating bends; $K_{pn}$ is the springback curvature after reciprocating bending *n* times; $K_{1}$ is the curvature after the first bending; $K_{0}$ is the initial curvature; $M_{1}$ is the loading moment of the first bending; $K_{n}$ is the curvature after reciprocating bending *n* times; $K_{{p\left( {n - 1} \right)}}$ is the springback curvature after reciprocating bending *n*-1 times; $M_{n}$ is the loading moment of the *n*th bending; *I* is the moment of inertia of the pipe cross-section, $I = \frac{\pi }{4}\left( {R_{1}^{4} - R_{2}^{4} } \right)$, $R_{1}$ is the inner diameter of the pipe, $R_{2}$ is the outer diameter of the pipe.

Based on small curvature plane bending springback theory, the springbck curvature of the pipe is deduced after reciprocating bending* n* times along the axial direction by using the mathematical induction method.

#### Straightness

When the first bending is reversed, the uniform equation for curvature after springback is

28 $$\begin{gathered} K_{{{\text{p}}n}} = K_{n} - \frac{D}{E}\left( {K_{n} - K_{n - 1} } \right) - \left( \frac{D}{E} \right)^{2} \left( {K_{n - 1} - K_{n - 2} } \right) - \ldots \hfill \\ - \left( \frac{D}{E} \right)^{n - 1} \left( {K_{2} - K_{1} } \right) + \left( { - 1} \right)^{n + 1} \left( {\frac{{16\sigma_{0} \left( {R_{1}^{3} - R_{2}^{3} } \right)}}{{3\pi \left( {R_{1}^{4} - R_{2}^{4} } \right)(E + D)}}} \right) \hfill \\ \end{gathered}$$

where, $K_{2}$ is the curvature after the second bending; $K_{n - 1}$ is the curvature after reciprocating bending *n*-1 times; $K_{n - 2}$ is the curvature after reciprocating bending *n*-2 times.

When the first bending is positive, the uniform equation for curvature after springback is

29 $$\begin{gathered} K_{{{\text{p}}n}} = K_{n} - \frac{D}{E}\left( {K_{n} - K_{n - 1} } \right) - \left( \frac{D}{E} \right)^{2} \left( {K_{n - 1} - K_{n - 2} } \right) - \ldots \hfill \\ - \left( \frac{D}{E} \right)^{n - 1} \left( {K_{2} - K_{1} } \right) + \left( { - 1} \right)^{n} \left( {\frac{{16\sigma_{0} \left( {R_{1}^{3} - R_{2}^{3} } \right)}}{{3\pi \left( {R_{1}^{4} - R_{2}^{4} } \right)(E + D)}}} \right) \hfill \\ \end{gathered}$$

Equations (28) and (29) can be expressed uniformly as

30 $$\overline{K}_{{{\text{p}}n}} = f\left( {D,E,\sigma_{0} ,R_{1} ,R_{2} ,K_{1} ,K_{2} , \ldots K_{n} } \right)$$

Equation (30) proves that multiple reciprocating bending can annihilate the difference in the axial initial curvature of the pipe, so that the curvature can be unified to the same direction and value. The uniform curvature is related to the material properties, the inner and outer diameters of the pipe, and the bending curvature. From the above analysis, it can be seen that the straightening deformation process belongs to a small deformation. That is, when the loading curvature ${K}_{n}$ tends to 0, the axial curvature after reciprocating bending and springback is also close to 0. The straightening process is achieved.

#### Ovality

When the first bending is reversed, the uniform equation for curvature after springback is

31 $$\begin{gathered} K_{{{\text{p}}n}} = K_{n} - \frac{D}{E}\left( {K_{n} - K_{n - 1} } \right) - \left( \frac{D}{E} \right)^{2} \left( {K_{n - 1} - K_{n - 2} } \right) - \ldots \hfill \\ - \left( \frac{D}{E} \right)^{n - 1} \left( {K_{2} - K_{1} } \right) + \left( { - 1} \right)^{n + 1} \frac{{3\sigma_{0} }}{(E + D)t} \hfill \\ \end{gathered}$$

When the first bending is positive, the uniform equation for curvature after springback is

32 $$\begin{gathered} K_{{{\text{p}}n}} = K_{n} - \frac{D}{E}\left( {K_{n} - K_{n - 1} } \right) - \left( \frac{D}{E} \right)^{2} \left( {K_{n - 1} - K_{n - 2} } \right) - \ldots \hfill \\ - \left( \frac{D}{E} \right)^{n - 1} \left( {K_{2} - K_{1} } \right) + \left( { - 1} \right)^{n} \frac{{3\sigma_{0} }}{(E + D)t} \hfill \\ \end{gathered}$$

Equations (31) and (32) can be expressed uniformly as

33 $$\overline{K}_{{{\text{p}}n}} = f\left( {D,E,\sigma_{0} ,t,K_{1} ,K_{2} , \ldots K_{n} } \right)$$

Equation (33) proves that multiple reciprocating bending can annihilate the difference in the circumferential initial curvature of the pipe. As a result, the curvature is unified to the same direction and value. The uniform curvature is related to material properties, wall thickness, and bending curvature. The rounding deformation process belongs to a small deformation. That is, when the loading curvature gradually tends to a certain value, the circumferential curvature of the pipe after reciprocating bending gradually tends to be consistent. The rounding process is achieved.

## Roller-shape design

**Figure 5:**
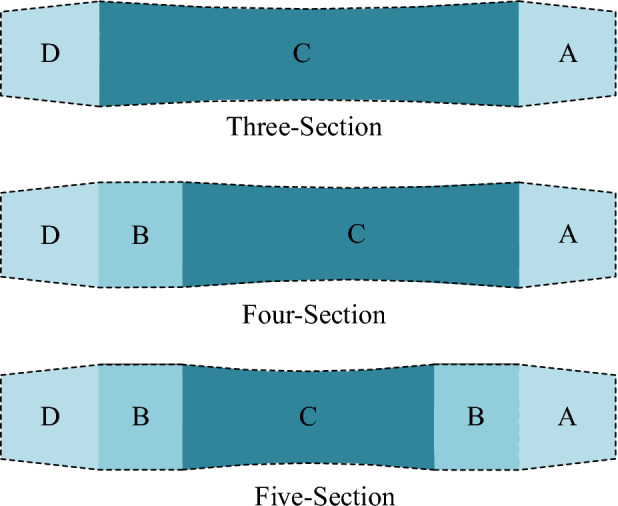
Roller-shape design scheme.

According to the “segmented” design idea and theoretical model, three roller-shape schemes are proposed. That is, “Three-Section” (loading section—ovality and straightness calibration section—unloading section), “Four-Section” (loading section—ovality calibration section—ovality and straightness calibration section—unloading section or loading section—ovality and straightness calibration section—ovality calibration section—unloading section), and “Five-Section” (loading section- ovality calibration section—ovality and straightness calibration section—ovality calibration section—unloading section), as shown in Fig. 5.

## Finite element model

**Figure 6:**
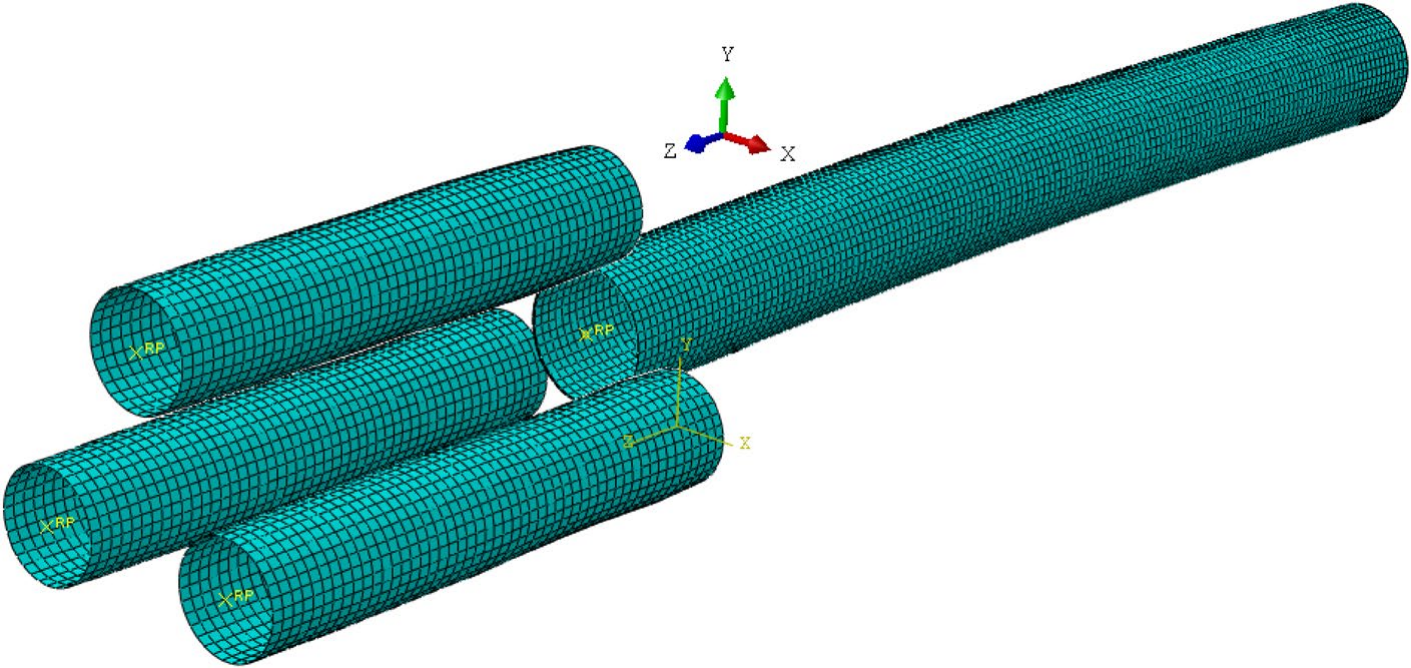
A finite element model of the “Four-Section” roller-shaped design.

The four finite element models for the four roller-shape designs are built using the ABAQUS 6.10 software package. Take the “Four-Section” roller-shape design as an example, as shown in Fig. 6. The geometric dimension of the pipe and roller and the mechanical properties of the pipe are shown in Tables 1, 2 and 3. According to the characteristics of the process, the pipe and the three rollers are modeled. Take the “Four-Section” roller-shape design as an example, an 8-node hexahedral linear uncoordinated mode element (C3D8R) is used to discretize the pipe. The total number of nodes is 31,020 and the number of units is 24,640. The three rollers are modeled as discrete rigid bodies. The contact between the pipe and the roller is set to pure master–slave contact and motion contact conditions, and the friction coefficient is 0.2. The speed of the pipe in the axial direction is 10 mm/s. The rotational speed of the two concave rollers is 6.28 rad/s.

**Table 1:** Geometric dimension of pipe.

Outer diameter $D_{p}$ (mm)	Length $L_{p}$ (mm)	Thickness $t$ (mm)	Initial ovality	Initial straightness
140	1500	2	5%	1%

**Table 2:** Mechanical properties of pipe.

Material	Elastic modulus *E* (GPa)	Yield stress $\sigma_{s}$ (MPa)	Plastic modulus *D* (MPa)
304	234	294	2842

**Table 3:** Geometric dimension of the rollers.

Outer diameter $D_{g}$ (mm)	Length $L_{g}$ (mm)	Taper of Section A (rad)	Taper of Section D (rad)	$K_{max}$ (/mm)	Roller shape curve of Section C
120	600	0.033	0.025	0.001	$0.16x^{2} - 0.0004y^{2} - 1 = 0$

## Results and discussion

**Figure 7:**
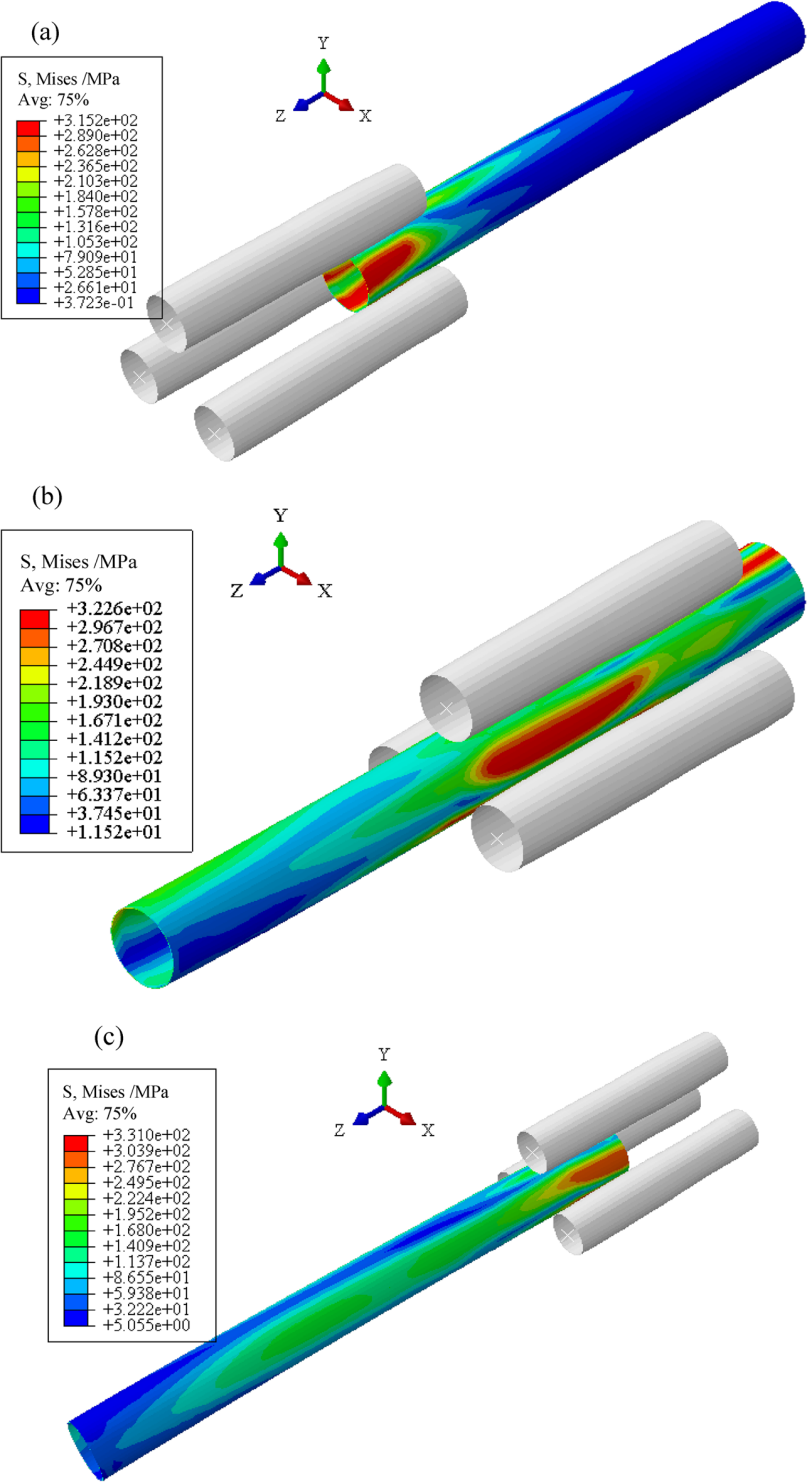
Equivalent stress distribution during this process.

Taking 304 pipes as the research object, the three-roller continuous and synchronous calibration process of ovality and straightness is analyzed. The equivalent stress distribution of the pipe during the process, as shown in Fig. 7. The equivalent stress on the inner and outer surfaces gradually increases and exceeds the yield stress of the material (294 MPa) during the propulsion of the pipe. Additionally, the circumferential and axial directions of the pipe continuously undergo reciprocating bending. The circumferential and axial curvature of the pipe are unified.

### Residual ovality

**Figure 8:**
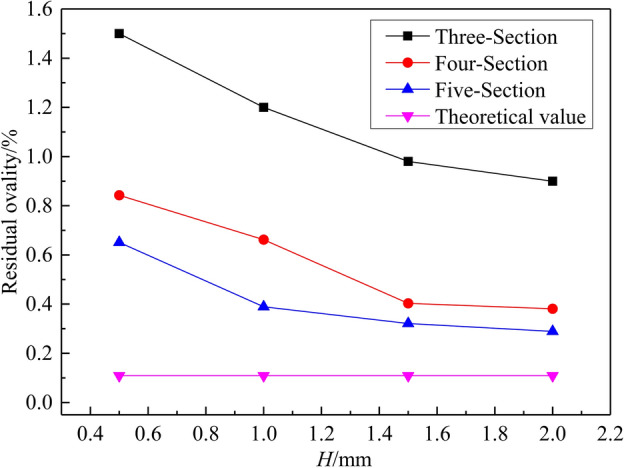
Results of residual ovality obtained by the different roller-shape designs. (The number of reciprocating bends *n* tends to be 140).

The results of residual ovality obtained by the different roller-shape designs are shown in Fig. 8. The residual ovality decreases gradually with the increase of the radial reduction *H*. When *H* reaches 1.5 mm, the “Five-Section” and “Four-Section” roller-shape schemes are closer to the theoretical values than the “Three-Section” scheme. The residual ovality can reach less than 0.4%, which meets the industrial requirements of large thin-walled pipes. The above results show that the straight section of the roller (section B) is more conducive to rounding than the variable curvature section (section C). The larger the proportion of section B, the smaller the residual ovality.

### Residual straightness

**Figure 9:**
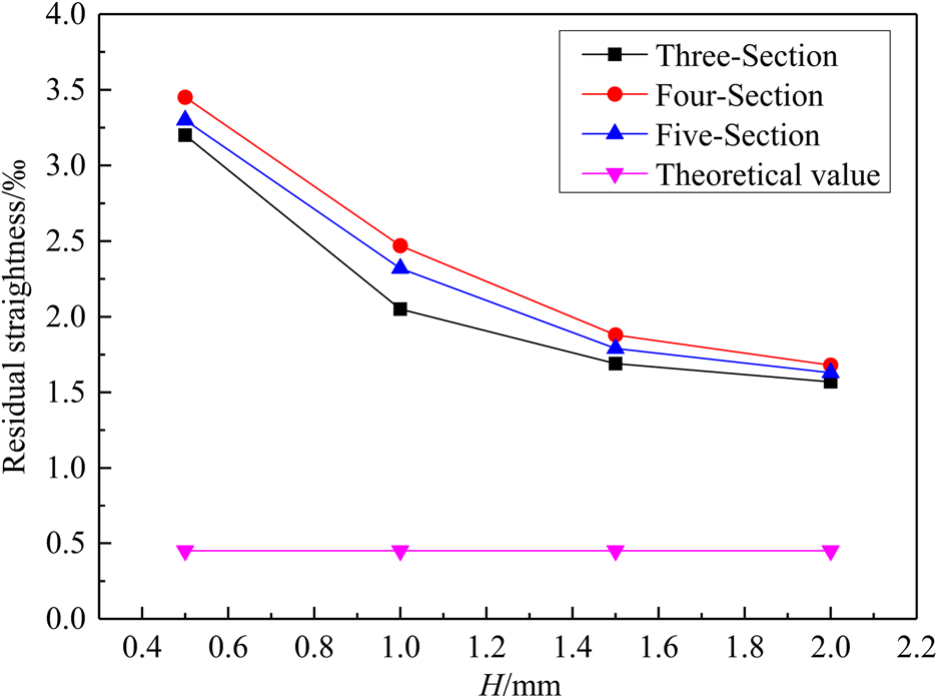
Results of residual straightness obtained by the different roller-shape designs. (The number of reciprocating bends *n* tends to be 140).

The results of residual straightness obtained by the different roller-shape designs are shown in Fig. 9. The residual straightness decreases gradually with the increase of the radial reduction *H*. The “Three-Section” roller-shape scheme is closer to the theoretical values than the “Four-Section” and “Five-Section” schemes. The residual straightness of three schemes can reach within 0.2%, which meets the industrial requirements of large thin-walled pipes. The above results show that the larger the proportion of section C, the smaller the residual straightness.

### Maximum residual stress

**Figure 10:**
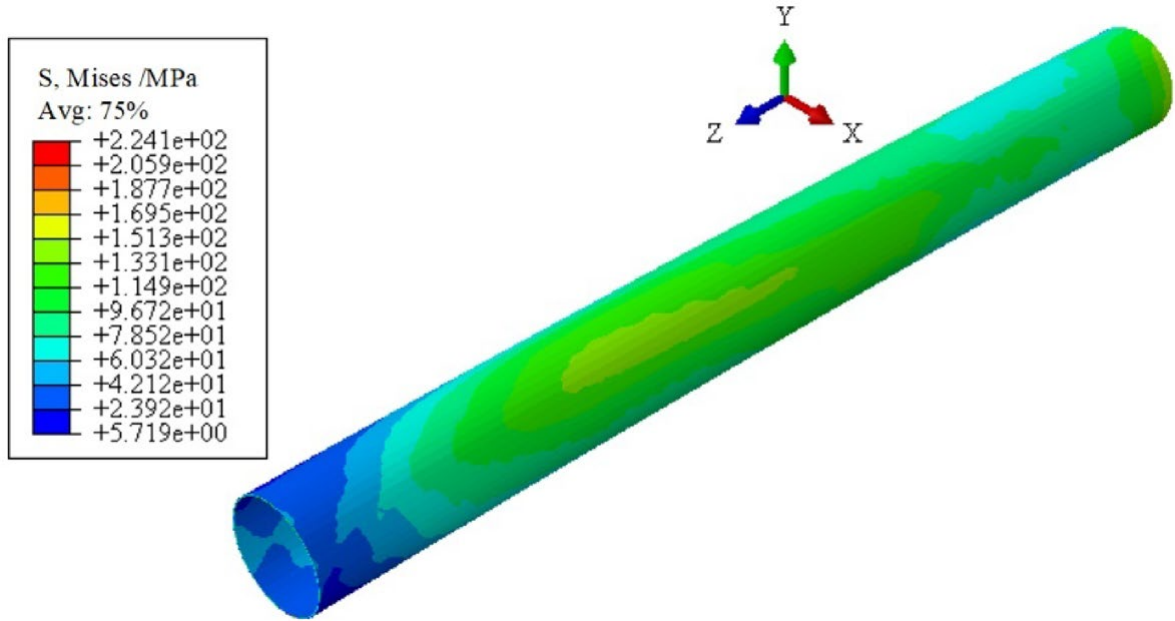
Equivalent stress distribution contour diagram of the straightened pipe. (“Four-Section” scheme; *H* is 1.0 mm).

Residual stress directly affects the service performance of large thin-walled pipes. Figure 10 is the equivalent stress distribution contour diagram of the straightened pipe of the “Four-Section” scheme when the *H* is 1.0 mm. The pipe is not unstable and does not undergo serious distortion. The main reason is the inconsistency of the deformation of the inner and outer of the pipe. The residual stresses are mainly concentrated at the outlet end of the pipe. The whole pipe is distributed in a ring shape, which gradually increases along the *z*-axis. By analyzing the distribution of the equivalent stress, the contact between the roller and the pipe can be inferred. It provides a reference for the correction of roller-shape design and the optimization of process parameters.

**Figure 11:**
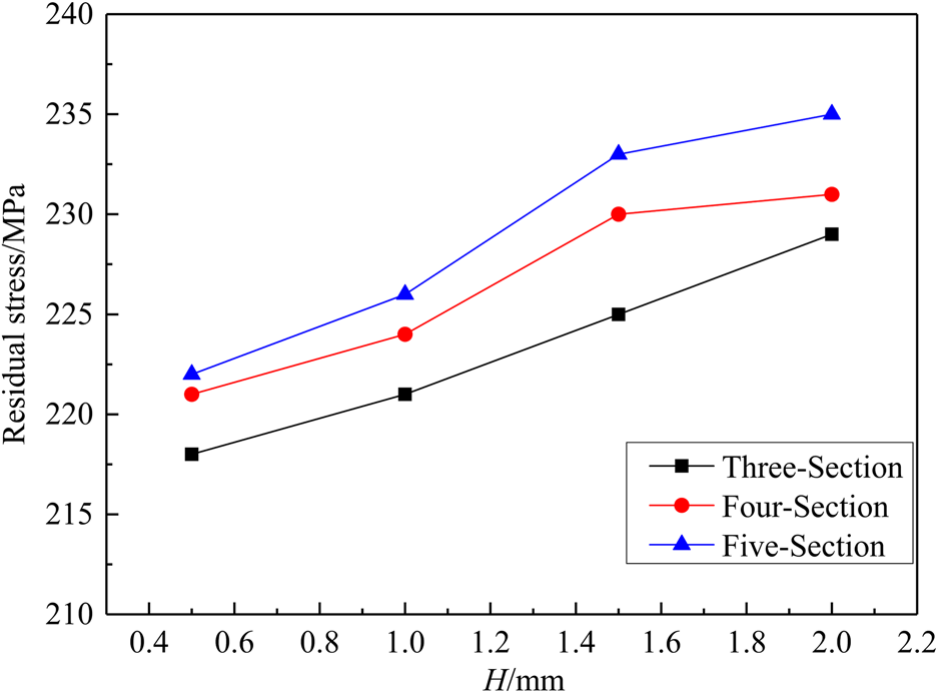
Results of residual stress obtained by the different roller-shape designs. (The number of reciprocating bends *n* tends to be 140).

The results of residual stress obtained by the different roller-shape designs are shown in Fig. 11. The residual stress increases gradually with the increase of the radial reduction *H*. When the same *H* is applied, the residual stresses of the three schemes do not differ by more than 10 MPa. The maximum residual stress shall not exceed 235 MPa.

## Experimental validations

**Figure 12:**
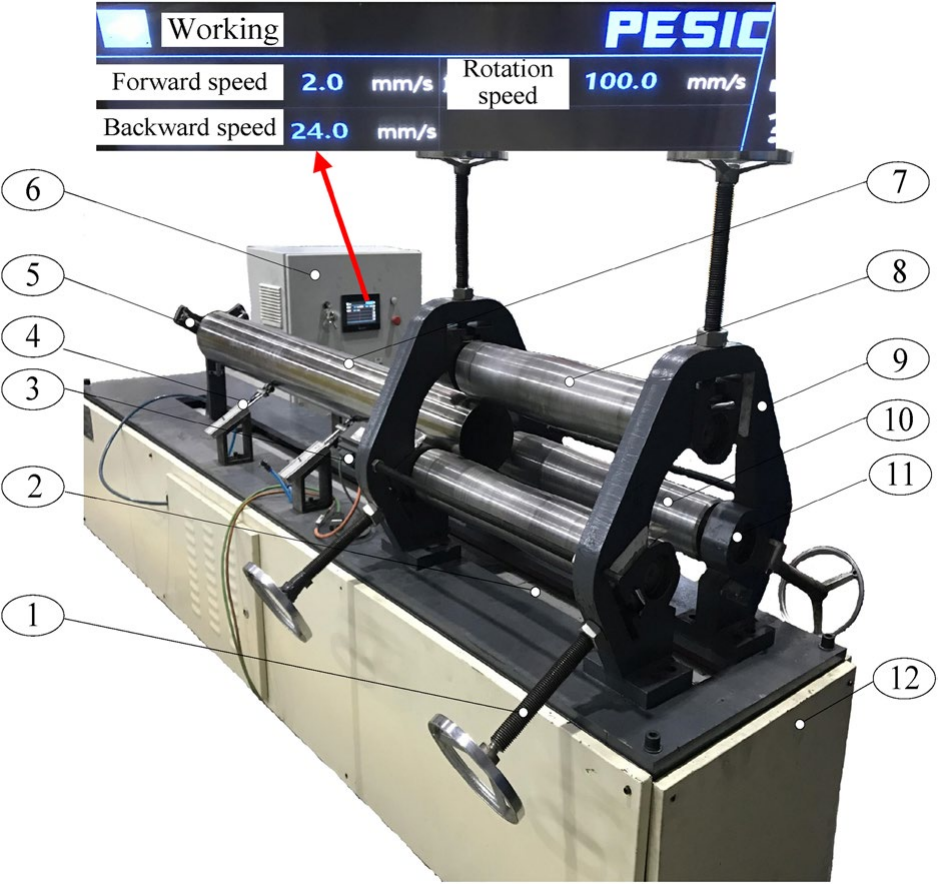
Experimental device for pipe calibration. 1, Screw. 2, Lead screw drive. 3, Servo motor. 4, Support assembly. 5, Push plate. 6, Control cabinet. 7, Pipe. 8, Upper roller. 9, Frame. 10, Lower roller. 11, Slider. 12, Pedestal.

The experimental device for pipe calibration is shown in Fig. 12. The device can realize both continuous rounding and continuous straightening of large thin-walled pipes. The geometric dimensions and mechanical properties of experimental pipes are shown in Table 4. The geometric dimension of experimental rollers is shown in Table 5.

**Table 4:** Geometric dimensions and mechanical properties of experimental pipes.

Material	Elastic modulus *E*/GPa	Yield stress $\sigma_{s}$/MPa	Plastic modulus *D*/MPa	Outer diameter *D*_p_/mm	Length *L*_p_/mm	Thickness *t*/mm	Initial ovality	Initial straightness
304	234	294	2842	140	1000	2/1.5	5%	1%

**Table 5:** Geometric dimension of experimental rollers.

Outer diameter $D_{g}$/mm	Length $L_{g}$/mm	Proportion of rollers	Taper of Section A /rad	Taper of Section D/rad	$K_{n}$ /(/mm)	Roller shape curve of Section C
120	600	1:2:4:2:1	0.033	0.025	0.001	$0.16x^{2} - 0.0004y^{2} - 1 = 0$

As shown in Fig. 12, the roller is connected with the slider fixed on the frame via bearing. The slider can slide vertically along the frame surface via a screw to adjust the radial reduction of the three rollers. The support assembly keeps the balance of the pipe. The servo motor drives two lower rollers to rotate synchronously, which drives the pipe and the upper roller to start turning. At the same time, the push plate drives the pipe to move along the slideway. So far, the calibration process of the pipe is realized.

The 3000iTM series portable coordinate measuring instrument produced by CimCore was used to obtain the coordinates of the points around the outer diameter of the pipe before and after deforming. The coordinates of these points were processed to obtain the straightness and ovality of the pipe.

**Figure 13:**
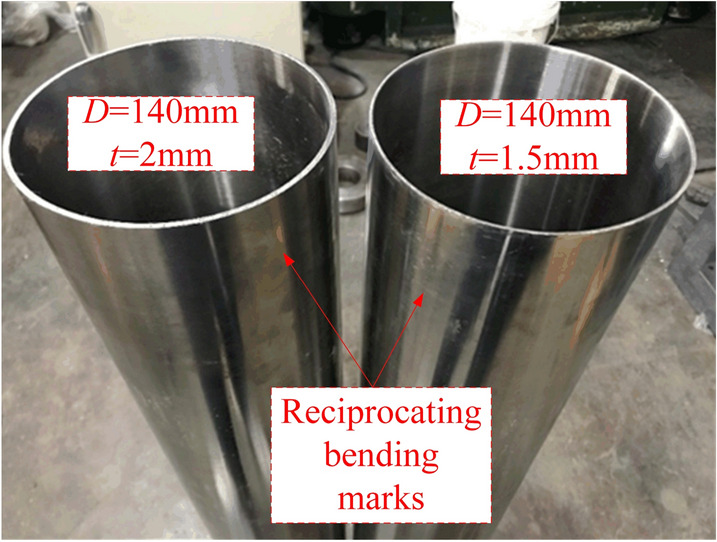
Forming effect of 304 stainless steel pipes.

As shown in Table 6, the ovality and straightness of the pipe are compared. It is found that the residual ovality can reach less than 1% and the residual straightness can reach less than 0.2%, which meets the industrial standard. The above proves the feasibility of the process. In the future, we will continue to use the simulation results to verify the calibration effect of the roller-shape scheme. Forming effect of 304 stainless steel pipes are shown in Fig. 13.

**Table 6:** Results after calibration.

Material	Outer diameter *D*_p_/mm	Thickness *t*/mm	Initial ovality/%	Initial straightness/%	Reduction *H*/mm		Residual ovality /%	Residual straightness /%
304	140	2.0 1.5	2.56 2.85	0.31 0.38		1.5 2.0	0.58 0.65	0.19 0.17

## Conclusions

1. A theoretical model for roller-shape design of three-roller continuous and synchronous adjusting straightness and ovality process for large thin-walled pipes is proposed. The model can provide a theoretical basis for presetting the process parameters and optimizing the roller-shape.
2. The residual ovality and residual straightness of pipes decrease with the increase of the radial reduction *H*. The larger the proportion of section B, the smaller the residual ovality. The larger the proportion of section C, the smaller the residual straightness. The maximum residual stress increases gradually with the increase of the radial reduction *H*.
3. The residual ovality can reach less than 1% and the residual straightness can reach less than 0.2%, which meets the industrial standard.

### Ethical approval

The authors declare that this manuscript was not submitted to more than one journal for simultaneous consideration. Also, the submitted work is original and not have been published elsewhere in any form or language.

### Consent to participate and publish

The authors declare that they participated in this paper willingly and the authors declare to consent to the publication of this paper.
